# Supplementary material for: A Formalized Method to Acclimate Dogs to Voluntary Treadmill Locomotion at Various Speeds and Inclines
Source: Animals (Basel). 2022 Feb 24;12(5):567. doi: 10.3390/ani12050567 (PMC8908820; doi:10.3390/ani12050567)
Supplement: Supplementary file 1 [file animals-12-00567-s001.zip › Text S1_ Penn Vet Working Dog Center Treadmill Acclimation Protocol.pdf]

Penn Vet Working Dog Center Treadmill Acclimation Protocol

Level	2% (flat) treadmill incline	10% treadmill incline	20% treadmill incline	Criteria time (sec)
1	0.5 mph (0.8 kph) - slow walk			5
2	0.5 mph (0.8 kph) - slow walk			30
3	1.0 mph (1.6 kph) - slow walk			30
4	2.0 mph (3.2 kph) - walk			30
5	3.0 mph (4.8 kph) - fast walk			30
6		0.5 mph (0.8 kph) - slow walk		30
7		1.0 mph (1.6 kph) - slow walk		30
8		2.0 mph (3.2 kph) - walk		30
9		3.0 mph (4.8 kph) - fast walk		30
10			0.5 mph (0.8 kph) - slow walk	30
11			1.0 mph (1.6 kph) - slow walk	30
12			2.0 mph (3.2 kph) - walk	30
13			3.0 mph (4.8 kph) - fast walk	30
14	4.0 mph (6.4 kph) - slow trot			30
15		4.0 mph (6.4 kph) - slow trot		30
16			4.0 mph (6.4 kph) - slow trot	30
17	5.0 mph (8.0 kph) - trot			30
18	6.0 mph (9.7 kph) - fast trot			30
19	7.0 mph (11.3 kph) - slow canter			30
20		5.0 mph (8.0 kph) - trot		30
21		6.0 mph (9.7 kph) - fast trot		30
22		7.0 mph (11.3 kph) - slow canter		30
23			5.0 mph (8.0 kph) - trot	30
24			6.0 mph (9.7 kph) - fast trot	30
25			7.0 mph (11.3 kph) - slow canter	30
