# Supplementary material for: A Formalized Method to Acclimate Dogs to Voluntary Treadmill Locomotion at Various Speeds and Inclines
Source: Animals (Basel). 2022 Feb 24;12(5):567. doi: 10.3390/ani12050567 (PMC8908820; doi:10.3390/ani12050567)
Supplement: Supplementary file 1 [file animals-12-00567-s001.zip › Text S2_ Penn Vet Working Dog Center Treadmill Acclimation Session Protocol.pdf]

Penn Vet Working Dog Center
Treadmill Acclimation Session Protocol

This protocol should be completed 2-3 times per week on nonconsecutive days. The room in which treadmill sessions are completed should have few distractions. A food reward that is easy for the dog to swallow while moving on the treadmill (e.g., frozen peanut butter and water mix) should be used. A toy reward may be used, but should only be given to the dog after successfully completing the desired level of treadmill acclimation and the treadmill has stopped. Reward the dog with the toy after the treadmill has stopped to prioritize the safety of the dog. Acclimation sessions should be performed in the morning or before other activities or training to maximize focus.

1. Assess the dog for soreness and record the results.
2. Perform the PVWDC FTW Warm-up [18].
    - On-leash walk for 30 seconds
    - On-leash trot for 30 seconds
    - Paws-up for 15 seconds
    - Figure-8 for 3 repetitions
3. Have the dog get on the treadmill.
    - If the dog has not completed level 5, skip to step 6.
4. Perform a warm-up on the treadmill at 0-2% incline.
    - If the dog has completed level 5, have them walk at 1.5 mph (2.4 kph) for approximately 2 minutes at 0-2% incline.
    - If the dog has completed level 7, have them walk at 3.0 mph (4.8 kph) for approximately 2 minutes at 0-2% incline.
5. Have the dog get off the treadmill for a quick break.
    - Slowly bring the treadmill to a stop with the dog still on it. Guide the dog down the back of the treadmill to not teach them they can jump off the side or when the treadmill is still moving.
    - Keep the dog off the treadmill for 30-60 seconds on a short leash so they cannot roam around or get distracted.
    - The break allows a dog to learn that they are only rewarded when they are on the treadmill, and the dog should be motivated to get back on the treadmill to obtain more of their reward.
6. Have the dog get on the treadmill and start them at their maximum level reached in the previous session minus two levels.
    - For example, if the dog reached level 14 in the last session, start at level 12, ensure the criteria for the level is met, move to level 13, and ensure the criteria for the level is met.
7. Complete the maximum level reached in the previous session.
8. Have the dog get off the treadmill for another break.
    - Keep the dog off the treadmill for 30-60 seconds on a short leash so they cannot roam around or get distracted.

    - Review and determine how the dog reacted behaviorally to this session. Did they show signs of stress? Did they seem motivated to continue?
9. If the dog was comfortable, have them get back on the treadmill and begin acclimating them on novel levels.
    - For example, if the dog reached level 14 in the last session and was comfortable at level 14 during this session, increase to level 15.
10. If the dog shows signs of physical or behavioral stress (See ‘Penn Vet Working Dog Center Treadmill Acclimation Assessment’ for signs of behavioral stress), decrease the speed and/or incline to where the dog is comfortable
    - This should be done at any point during the acclimation session.
    - Once the dog has shown they are comfortable, increase to a higher level and reassess if they are comfortable or uncomfortable.
11. Continue progressing the dog with new levels. Stop progressing when one of the following criteria is met:
    - The dog is consistently behaviorally uncomfortable doing the level and showing signs of stress (See ‘Penn Vet Working Dog Center Treadmill Acclimation Assessment’ for signs of behavioral stress).
    - The dog is not maintaining their position (side-to-side or front-to-back) on the treadmill
    - The dog has accumulated 10 minutes on the treadmill
12. If the dog has not reached level 5, have them get off the treadmill.
13. If the dog has reached level 5, perform a cool-down on the treadmill by having them walk at 1.5 mph (2.4 kph) for approximately 2 minutes. Have the dog get off the treadmill.
14. Perform the PVWDC FTW Cool-down [18].
    - On-leash walk for 30 seconds while observing for lameness and other abnormalities
    - Paws-up for 15 seconds
    - Four Position Cookie Stretch on each side
    - Paw, pad, and nail check

References

18. Farr, B.D., M.T. Ramos, and C.M. Otto, *The Penn Vet Working Dog Center Fit to Work Program: A Formalized Method for Assessing and Developing Foundational Canine Physical Fitness.* Frontiers in Veterinary Science, 2020. **7**(470).
