# Supplementary material for: A Formalized Method to Acclimate Dogs to Voluntary Treadmill Locomotion at Various Speeds and Inclines
Source: Animals (Basel). 2022 Feb 24;12(5):567. doi: 10.3390/ani12050567 (PMC8908820; doi:10.3390/ani12050567)
Supplement: Supplementary file 1 [file animals-12-00567-s001.zip › Text S3_ Penn Vet Working Dog Center Endurance Soreness Assessment.pdf]

Penn Vet Working Dog Center Endurance Soreness Assessment

______________________’s Soreness Record

Date	Epaxials		Gluteals		Iliopsoas		Cranial thigh		Caudal thigh		Total Tension Score	Resistance to movements	Evaluator’s Initials & Notes
	L	R	L	R	L	R	L	R	L	R			
	H 0 1 2	H 0 1 2	H 0 1 2	H 0 1 2	H 0 1 2	H 0 1 2	H 0 1 2	H 0 1 2	H 0 1 2	H 0 1 2		P-u 8s	
	H 0 1 2	H 0 1 2	H 0 1 2	H 0 1 2	H 0 1 2	H 0 1 2	H 0 1 2	H 0 1 2	H 0 1 2	H 0 1 2		P-u 8s	
	H 0 1 2	H 0 1 2	H 0 1 2	H 0 1 2	H 0 1 2	H 0 1 2	H 0 1 2	H 0 1 2	H 0 1 2	H 0 1 2		P-u 8s	
	H 0 1 2	H 0 1 2	H 0 1 2	H 0 1 2	H 0 1 2	H 0 1 2	H 0 1 2	H 0 1 2	H 0 1 2	H 0 1 2		P-u 8s	
	H 0 1 2	H 0 1 2	H 0 1 2	H 0 1 2	H 0 1 2	H 0 1 2	H 0 1 2	H 0 1 2	H 0 1 2	H 0 1 2		P-u 8s	
	H 0 1 2	H 0 1 2	H 0 1 2	H 0 1 2	H 0 1 2	H 0 1 2	H 0 1 2	H 0 1 2	H 0 1 2	H 0 1 2		P-u 8s	
	H 0 1 2	H 0 1 2	H 0 1 2	H 0 1 2	H 0 1 2	H 0 1 2	H 0 1 2	H 0 1 2	H 0 1 2	H 0 1 2		P-u 8s	
	H 0 1 2	H 0 1 2	H 0 1 2	H 0 1 2	H 0 1 2	H 0 1 2	H 0 1 2	H 0 1 2	H 0 1 2	H 0 1 2		P-u 8s	

Abbreviations: heat (H), Paws-up (P-u), and Figure-8s (8s)
Signs of soreness: resistant to perform movements, muscle tension, behavioral response to palpation, warmth
Scoring: 0 - no soreness, 1 - mild soreness, 2 - moderate or greater soreness
Response: If a soreness of 2 is identified in any area or a total score of 4 or greater, complete the PVWDC FTW Warm-up, perform a 5:00 walk or trot (as appropriate for the dog’s acclimation stage) on the treadmill with no incline, and complete the PVWDC FTW Cool-down [18].

References

18. Farr, B.D., M.T. Ramos, and C.M. Otto, *The Penn Vet Working Dog Center Fit to Work Program: A Formalized Method for Assessing and Developing Foundational Canine Physical Fitness.* Frontiers in Veterinary Science, 2020. **7**(470).
