# Supplementary material for: A Formalized Method to Acclimate Dogs to Voluntary Treadmill Locomotion at Various Speeds and Inclines
Source: Animals (Basel). 2022 Feb 24;12(5):567. doi: 10.3390/ani12050567 (PMC8908820; doi:10.3390/ani12050567)
Supplement: Supplementary file 1 [file animals-12-00567-s001.zip › Text S4_ Penn Vet Working Dog Center Treadmill Acclimation Assessment.pdf]

Penn Vet Working Dog Center
Treadmill Acclimation Assessment

The assessment ends when one of the following criteria are met:

- The dog finishes the protocol (can complete the maximum level of 30 seconds at 7 mph (11.3 kph) on 20% incline)
- The dog demonstrates they are physically unable to complete a level by:
  - Being unable to maintain their position at the front of the treadmill for more than 5 seconds despite all attempts to motivate them (e.g., verbal reinforcement or presenting food reward)
  - Stumbling or deviating laterally on the running surface
- The dog demonstrates they are behaviorally uncomfortable by:
  - Making rapid head movements and looking over at the side of the treadmill (they are thinking of jumping off)
  - Showing a rapid change in respiratory rate (sign of behavioral stress rather than physical stress)
  - Unwilling to take food reward (note: some dogs, however, will be unwilling to take food when it becomes physically challenging)
  - Showing avoidance behavior to the treadmill or handler (when off the treadmill)

Acclimation assessment protocol

- Perform the PVWDC FTW Warm-up [18]
- 0-2% incline (treadmill on flat ground)
  - 2:00 at 2 mph (3.2 kph) (warm-up)
  - 0:30 at 3 mph (4.8 kph)
  - 0:30 at 4 mph (6.4 kph) (if physically capable)
  - 0:30 at 5 mph (8.0 kph) (if physically capable)
  - 0:30 at 6 mph (9.7 kph) (if physically capable)
  - 0:30 at 7 mph (11.3 kph) (if physically capable)
  - Stop the treadmill
  - Let the dog relax for 1:00 and drink water
  - Only progress to 10% incline if the dog is behaviorally and physically comfortable at all paces
- 10% incline
  - 0:30 at 2 mph (3.2 kph)
  - 0:30 at 3 mph (4.8 kph)
  - 0:30 at 4 mph (6.4 kph) (if physically capable)
  - 0:30 at 5 mph (8.0 kph) (if physically capable)
  - 0:30 at 6 mph (9.7 kph) (if physically capable)
  - 0:30 at 7 mph (11.3 kph) (if physically capable)
  - Stop the treadmill
  - Let the dog relax for 1:00 and drink water

    - Only progress to 20% incline if the dog is behaviorally and physically comfortable at all paces
- 20% incline
    - 0:30 at 2 mph (3.2 kph)
    - 0:30 at 3 mph (4.8 kph)
    - 0:30 at 4 mph (6.4 kph) (if physically capable)
    - 0:30 at 5 mph (8.0 kph) (if physically capable)
    - 0:30 at 6 mph (9.7 kph) (if physically capable)
    - 0:30 at 7 mph (11.3 kph) (if physically capable)
    - 2:00 at 1.5 mph (2.4 kph) (cool-down)
    - Stop the treadmill
    - Let the dog relax for 1:00 and drink water
- Perform the PVWDC FTW Cool-down [18]
- The dog is considered acclimated to the highest progression they completed for 0:30 while demonstrating behavioral and physical comfort.
- The dog has completed the acclimation assessment if they are behaviorally comfortable for 0:30 at all paces and inclines up to the limit of their physical ability

References

18. Farr, B.D., M.T. Ramos, and C.M. Otto, *The Penn Vet Working Dog Center Fit to Work Program: A Formalized Method for Assessing and Developing Foundational Canine Physical Fitness.* Frontiers in Veterinary Science, 2020. **7**(470).
